# Efficient Measurement of Length Distribution of 1D Nanoparticles in Solution *via* Optical Polarimetry

**DOI:** 10.1021/acsnano.5c14994

**Published:** 2025-12-01

**Authors:** Richard J. Castellano, Da-Chi Yang, Sei Jin Park, Pavel Shapturenka, Robert F. Praino, Jeffrey A. Fagan, Francesco Fornasiero, Jerry W. Shan

**Affiliations:** † Department of Mechanical & Aerospace Engineering, Rutgers, 242612 The State University of New Jersey, New Brunswick, New Jersey 08854, United States; ‡ CHASM Advanced Materials, Inc., Canton, Massachusetts 02021, United States; § Physical and Life Sciences Directorate, 4578 Lawrence Livermore National Laboratory, Livermore, California 94550, United States; ∥ Materials Science and Engineering Division, National Institute of Standards and Technology, Gaithersburg, Maryland 20899, United States

**Keywords:** carbon nanotubes, 1D nanoparticles, optical polarimetry, linear dichroism, electric field alignment, length measurement, suspension characterization

## Abstract

The efficient measurement of the length distribution of nanotubes, nanowires, and other one-dimensional (1D) nanoparticles in solution is important to enable their incorporation into materials and devices and to optimize their processing for properties of interest, such as thermal/electrical conductivity or mechanical strength, in suspensions and composites. We report an electric-field (*E*-field)-assisted optical-polarimetry technique to measure the length distribution of ensembles of high-aspect-ratio particles in dilute suspension. The degree of alignment of polarizable 1D particles suspended in a fluid under Brownian motion explicitly depends on the *E*-field strength and the particle length. We show that it is possible to extract the length distribution of 1D nanoparticles suspended in an insulating fluid by applying a range of *E*-fields and using optical polarimetry to measure the corresponding alignment order parameter. Notably, the method is relatively insensitive to the diameter of the 1D particles, which can be poorly known or vary within a sample. The technique is validated with silver nanowires and carbon nanotubes of known lengths, as well as polymer-depletion-length-separated single-wall carbon nanotube samples with length distributions independently measured with analytical ultracentrifugation. Finally, we demonstrate the ability of the optical-polarimetry technique to quantify changes in the length distribution of ultranarrow, sub-nanometer-diameter single-wall carbon nanotubes under different types and durations of ultrasonication. Within its range of applicability (polarizable 1D nanoparticles in the 0.5 to 15 μm length range, constrained by the voltage stability of the media and the suspended particles), the *E*-field-assisted optical-polarimetry method is a particularly efficient and accurate method to measure the length distribution of nanowires and nanotubes in suspension.

## Graphical abstract

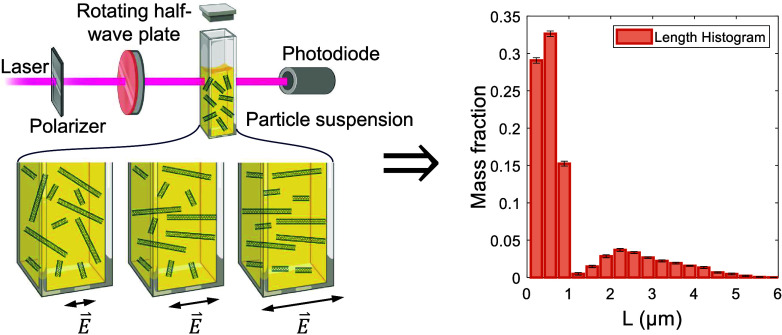

Accurate and efficient characterization of the length distributions of one-dimensional (1D) nanomaterials such as nanotubes and nanowires in suspension is crucial to tailoring devices and products that extract the full macroscopic benefit of the nanomaterial properties. For instance, the electrical, thermal, and mechanical properties of carbon nanotube (CNT) composites are dramatically affected by the length and aspect ratio of the constituent CNTs.^1–3^ Additionally, the viscosity^4^ and stability^5^ of CNT suspensions are a strong function of CNT length, and the use of well-dispersed CNT suspensions also strongly affects the properties of the resulting composite. In particular, for membrane applications in which aligned nanotubes serve as pores for mass transfer,^6–22^ it is essential that the nanotubes be longer than the membrane. However, nanotubes much longer than the membrane thickness can reduce membrane permeability due to the lower concentration that is required to avoid entanglement during field-induced alignment and create stable suspensions.^18,23^ Measurement methods that allow rapid characterization of the length distribution of 1D nanoparticles in solution from a statistically significant sample size are highly desirable to guide and accelerate materials design.

Existing techniques to characterize the lengths of 1D nanomaterials are laborious, making it challenging to measure length distributions, particularly for solution-based samples that are utilized in many applications. Direct-imaging methods of observing individual particles, either through individual particle tracking^24–28^ or imaging, *e.g.*, scanning electron microscopy (SEM),^29^ atomic force microscopy (AFM),^30–34^ or transmission electron microscopy (TEM),^35^ are straightforward but limited in their ability to measure a great enough number of particles to provide robust statistics. These techniques also typically do not measure *in situ* length distributions of particles dispersed in solution. Other ensemble-length-distribution measurement methods like dynamic light scattering (DLS)^36,37^ and multiangle DLS^38–41^ must extract length and diameter data from the same measurements, making it difficult to observe variances in lengths and diameters separately. Laser diffraction,^42,43^ small-angle X-ray scattering,^44,45^ and small-angle neutron scattering^33,46–49^ have also been used to characterize CNTs, but the scattering has only been shown to qualitatively determine CNT length. Other solution-based techniques, namely viscosity and rheology measurements,^41,50–52^ flow-field fractionation,^53–55^ chromatography,^31,33,39,56–59^ analytical ultracentrifugation (AUC),^43,60–70^ and optical absorbance^38,71,72^ are promising but are constrained to measuring particle lengths below ≈ 1 μm, or the interpreted length scales with particle aspect ratio (thus necessitating prior knowledge of particle diameter for accurate length characterization).

In this work, we report an efficient optical technique to measure the length distribution of suspended, electrically polarizable 1D nanoparticles in the length range of 0.5 to 15 μm. Briefly, particles with polydisperse lengths are exposed to an alternating electric field (*E*-field) of increasing strength, reaching different degrees of alignment due to the competition between electrostatic forces and Brownian motion. The degree of alignment of the sample of 1D nanoparticles is quantified *via* linear dichroism,^4^ ,^73^ *i.e.*, differential absorption of linearly polarized light with orientation. Importantly, because the measured absorption anisotropy depends strongly on particle length but only weakly on particle diameter, the length-distribution measurement is robust to uncertainties or variation in the particle diameter. While other works use *E*-field-assisted optical methods to quantify the degree of *E*-field alignment^4,73^ and particle length by thermal relaxation time scales,^74,75^ this is the first work to measure particle length by probing the trend of degree-of-alignment with *E*-field strength.

## Results and Discussion

### Electric-Field Alignment

Polarizable 1D nanoparticles in fluid suspension develop an induced dipole *p⃗* under an applied *E*-field and tend to align with the field due to an electrostatic torque *p⃗* × *E⃗*. For nanoparticles, the *E*-field-induced alignment is opposed by thermal fluctuations of the suspending fluid. The Maxwell–Wagner interfacial polarization model^23,76–86^ shows that the induced dipole leading to alignment develops whenever the particle and fluid have either different electrical conductivities or permittivities. As detailed in the Supporting Information, the electrostatic potential energy *U* depends strongly on the nanoparticle length *L*, and only weakly on particle diameter *d*, namely, as $U\propto L^{3}/\left(\ln\frac{2L}{d}-1\right)$ . For the particles of this contribution and many more 1D nanoparticles, the assumption that all particles are highly polarizable relative to the surrounding fluid is accurate (see the Supporting Information for more details). Figure 1(a) illustrates that for a weak *E*-field that produces electrostatic orienting potentials for each length *U*(*L*) much smaller than the thermal energy *k*_B_ *T*, both short and long particles will be randomly oriented under the disturbance of Brownian motion. For a medium *E*-field, longer particles will align strongly, while shorter particles will remain more randomly oriented. Finally, for strong *E*-fields, *i.e.*, those for which the electrostatic orienting potential for all 1D nanoparticles is much larger than the thermal energy, both long and short particles will be highly aligned. Thus, we can quantify the length distribution of 1D nanoparticles in suspension if we can measure their degree of alignment under varying *E*-fields.

**Figure 1:**
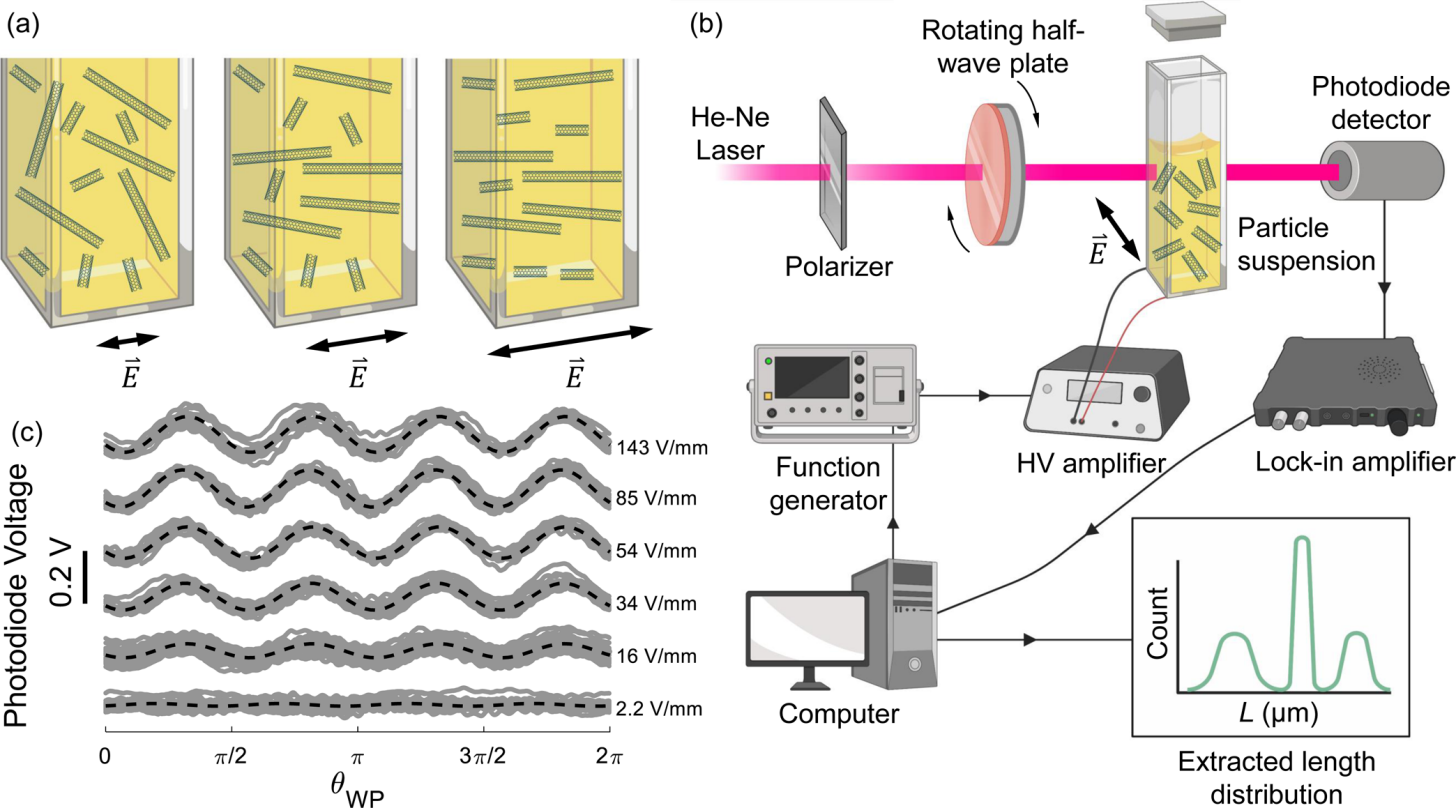
Schematic of the length-measurement system and typical signals. (a) Illustration of the alignment response of short and long particles. At low *E*-fields, the particles are randomly aligned. As the *E*-field strength increases, the longer particles align, while the shorter particles are still in a random alignment. At even higher *E*-fields, both the short and long particles are aligned. (b) Schematic of the *E*-field-alignment and optical-polarimetry system to observe the absorption anisotropy of particle suspensions as a function of an applied *E*-field. (c) Example of the recorded dichroism response of SWCNTs as a function of the half-wave plate angle θ_WP_. Photodiode voltages are offset in the *y*-axis by an arbitrary amount for better visibility. For lower voltages, the dichroism increases with *E*, and for *E* > 54 V/mm, the dichroism amplitude is seen to reach a saturation level. Parts of figure were created with BioRender.com.

### Measuring Alignment with Optical Polarimetry

After an applied *E*-field causes the polarizable particles in suspension to rotate into alignment, the aligned 1D particles will preferentially absorb incident light that is polarized in the direction of their long axis.^73,87,88^ To measure the degree of particle alignment, we pass linearly polarized light, with a rotating polarization angle, through the particle suspension during the application of an *E*-field and measure the transmitted intensity with a photodiode, as seen in Figure 1(b). We temporally vary the polarization of incident light with a rotating half-wave plate to rapidly probe the dichroism of the 1D nanoparticle suspension for all linear-polarization directions.^4^ This method allows for decoupling from artifacts, *e.g.*, baseline signal drift. More highly aligned 1D nanoparticle ensembles show greater absorption anisotropy in the form of optical dichroism. By observation of the dichroism increase with *E*-field magnitude up to alignment saturation, it is possible to extract the length distribution of particles in the sample.

An example set of recorded dichroism signals of a single-walled carbon nanotube (SWCNT) suspension can be seen in Figure 1(c). The intensity of the transmitted linearly polarized signal is graphed as a function of the rotation angle of the half-wave plate θ_WP_ for various magnitudes of the *E*-field. Because, as rods, the aligned particles absorb light with 180° symmetry and the angle of polarization cycles at twice the rotation rate of the half-wave plate, θ̇_WP_, the resulting dichroism signal oscillates at 4θ̇_WP_, which is apparent in Figure 1(c). At an *E*-field of 2.2 V/mm, the absorption anisotropy is hardly visible, while the optical dichroism increases for *E*-fields from 16 to 54 V/mm, and finally saturates as *E* increases beyond 54 V/mm.

As detailed in the Supporting Information, the dichroism amplitude is extracted from the raw signal by taking the magnitude of the Fourier-transformed photodiode signal at 4θ̇_WP_ for a given field strength *E*. The extracted absorption anisotropies are graphed as dashed lines in Figure 1(c). The unique dichroism signature of each individual length enables extraction of the length distribution by fitting the measured dichroism as a function of the applied field strength. The dichroism signal was modeled as a linear superposition of contributions from the constituent lengths, each scaled by an absorbance amplitude assumed to be directly proportional to the mass fraction at that length. An *E*-field frequency of 3000 Hz was selected to mitigate any charging or electrochemical reactions.

### Comparison to Existing Methods

To validate the optical-polarimetry technique, we made a detailed comparison between the optical-polarimetry technique described here and other common methods using a variety of samples of 1D nanoparticles, including silver nanowires and carbon nanotubes of different dimensions, grown with chemical vapor deposition^89–91^ (CVD) or with a cobalt–molybdenum catalyst^92,93^ (CoMoCAT), as detailed in Table 1. The carbon nanotubes were either grown to nominally uniform lengths in aligned forests on silicon wafers or length-fractionated by polymer-depletion-length-separation (PDLS). The length distributions of these samples were independently measured by direct SEM imaging, optical microscopy measurements, or AUC measurements. Where shown, uncertainties are reported as 1 standard deviation.

**Table 1:** Summary of Nanomaterial Samples Used to Compare Measurements from the Optical-Polarimetry Technique to Existing Measurement Methods

**sample label**: description	length	individual particle diameter	average bundle diameter
**AgNW**: Ag nanowires, PL-AgW100, PlasmaChem GmbH^a^	5 to 50 μm^d^	134 nm^f^, 100 nm^d^	individual^d^,^f^
**G1**: Uniform bundles of SWCNTs, grown by CVD on Si wafer^a^,^b^	6 to 8 μm^d^	1.79 nm^g^	242 nm^f^
**G2**: Uniform bundles of SWCNTs, grown by CVD on Si wafer^a^,^b^	8.3 μm^d^	2.39 nm^g^	331 nm^f^
**G3**: Uniform bundles of SWCNTs, grown by CVD on Si wafer^a^,^b^	12 to 13 μm^d^	2.32 nm^g^	263 nm^f^
**Sediment**: Individual CoMoCAT SWCNTs, longer sediment fraction from PDLS^c^	≈1.5 μm^e^	0.8 nm^h^	individual^d^
**Supernatant**: Individual CoMoCAT SWCNTs, shorter supernatant fraction from PDLS^c^	≈1.0 μm^e^	0.8 nm^h^	individual^d^

a Compared to SEM.

b Compared to optical microscopy.

c Compared to AUC.

d As described by supplier or manufacturer.

e Measured only in this work.

f Measured by SEM.

g Measured by TEM.

h Measured by optical fluorescence spectroscopy.

For optical-polarimetry measurements, the Ag nanowires were dispersed in hexadecane using bath sonication at a dilute concentration of 3 mg/L, a low concentration required to mitigate any particle–particle interaction under the *E*-field. At this concentration, the laser should pick up about 1.14 × 10^6^ Ag nanowires, which should provide robust statistics. Figure 2(a) shows the representative measured absorption anisotropy for the silver nanowires. The dichroism signal shows the expected increase and eventual saturation as an increasing fraction of the nanowires (starting with the longest ones) become aligned with increasing field strength. Figure 2(c) shows the measured length distributions for the Ag nanowires extracted from optical polarimetry. For validation, we also dried a sample of the Ag nanowires on a Si wafer and measured the length distribution with SEM. Comparison with SEM measurement for the Ag nanowires shows excellent agreement, with a peak centered at 1 to 2 μm and similar distribution shapes for both the optical-polarimetry and SEM measurements. We note that both measurements show a significantly shorter length than the nominal dimensions of the Ag nanowires. This is likely due to breakage of the Ag nanowires during processing, as well as settling of the longer, heavier Ag nanowires so that they did not appear in the samples used for the measurements. Nevertheless, clear consistency is seen between the optical-polarimetry and SEM measurements for the Ag nanowires.

**Figure 2:**
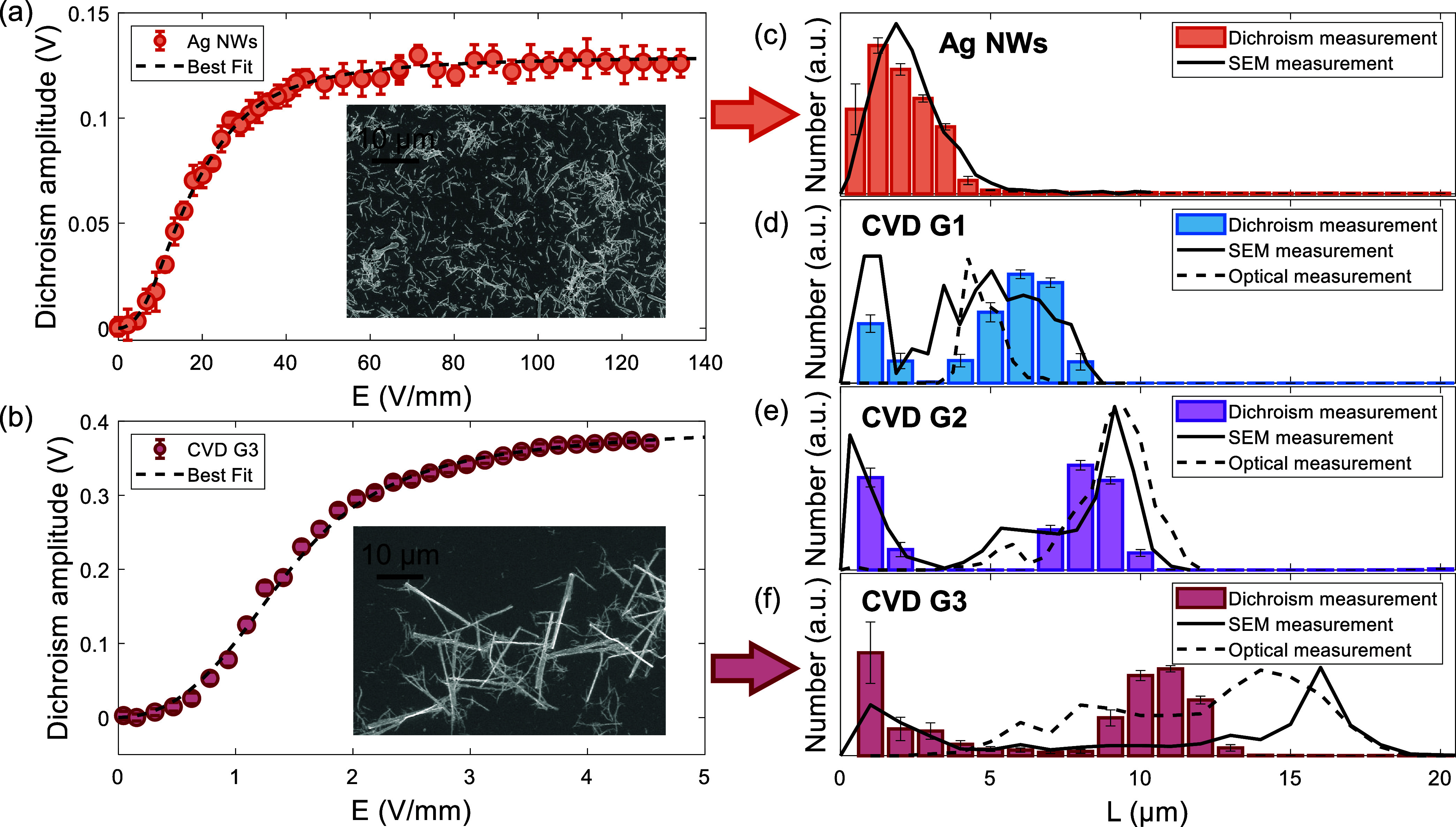
Validation of the technique with 1D nanoparticles of known dimensions. (a) Dichroism response of Ag nanowires as a function of the applied *E*-field. The units of the dichroism amplitude are the amplitude of the sine-wave voltage of the photodiode. Inset: SEM image of the Ag nanowires dried on a Si wafer. (b) Dichroism amplitude response of CVD SWCNTs from G3 as a function of the applied *E*-field. Inset: SEM image of a CVD SWCNT dispersion dried on a Si wafer. These CVD SWCNTs are bundled due to their growth in a dense, vertically aligned CNT forest. (c) Extracted length distribution of Ag nanowires compared to the SEM measurements. Error bars show standard deviation from 10 best fits of the dichroism data starting from random initial conditions, as described in the Supporting Information. (d–f) Extracted length distributions of CVD SWCNTs from the three samples.

For the next validation, we examined three samples (CVD growths G1, G2, and G3) of SWCNT forests grown to different nominally uniform lengths on Si wafers (additional details in the Supporting Information). The nanotubes were detached from the silicon wafer and dispersed in 1,2-dichlorobenzene (DCB) at dilute concentrations of 0.5 mg/L using mild bath sonication, and measured with the optical-dichroism method. The CVD-grown SWCNT samples exist primarily as bundles due to their growth in dense, vertically aligned forests. SEM images in Figure 2(b) inset confirm bundle diameters of up to 1 μm, significantly larger than individual tube diameters of ≈2 to 3 nm. Because a SWCNT bundle behaves as a single, rigid, electrically conductive particle in suspension, it is the bundle length that is measured by the optical-polarimetry method. Dichlorobenzene was chosen as the solvent for CNTs because it is effective in dispersing carbon nanotubes,^94–96^ and has a low electrical conductivity that allows the application of high *E*-fields without significant heating.

Figure 2(b) shows a representative optical-polarimetry response of the 12 to 13 μm long CVD SWCNTs from G3, along with SEM images of the nanotubes dried on silicon wafers. The dichroism amplitude shows the expected three regimes of the response, with initial low response, increase, and saturation with an increasing *E*-field strength. Of note, the response begins to saturate at an order-of-magnitude lower field strength than that of the Ag nanowires. This is because of the longer lengths of the SWCNTs, which increase their dipole moment and alignment torque at a given *E*-field. Figure 2(d–f) shows the dichroism-measured length distributions for the nominally uniform SWCNT forests of CVD samples G1 to G3. For validation, the length distributions measured by SEM and optical microscopy are also shown. Both dichroism and SEM show that the SWCNT forests have bimodal length distributions, with one peak at their nominal as-grown lengths and a second peak below 1 μm due to breakage of the SWCNTs by sonication. In general, the measured distributions agree well between the optical-polarimetry technique, SEM, and optical microscopy, although optical microscopy could not resolve lengths below approximately 0.5 to 1 μm due to the diffraction limit of optical imaging. Note that we hypothesize the discrepancy between the lengths measured by optical-polarimetry and SEM for G3 is most likely attributable to imaging samples that have been dried on a substrate. In preparing such SEM samples, there is an inherent dilemma: lower deposition density makes measurements inefficient, whereas higher deposition density increases the likelihood of overlapping or bundling and obscuring the true length distribution.

As a final validating comparison, we examined SWCNTs synthesized utilizing the CoMoCAT technique under conditions aimed at the selective production of (6, 5) chirality.^93,97^ Following synthesis, these CNTs were suspended in water and underwent several purification steps, finalizing with length-based fractionation through PDLS.^98^ This resulted in two distinct length fractions: shorter SWCNTs present in the supernatant and longer SWCNTs located in the sediment. Subsequently, these samples were transferred from the aqueous surfactant system to DCB using isopropanol (IPA) to strip the surfactant in a solvent-transfer process similar to an established method^99^ (see the Supporting Information). These surfactant-free individualized SWCNTs in DCB were examined for dichroism at concentrations of 5 × 10^–7^ g/L, and suspension stability was verified by consistent, repeated *E*-field sweeps for each sample. The two fractions of SWCNTs in DCB were compared to AUC measurements conducted at the National Institute of Standards and Technology (NIST)^66,67^ on the aqueous dispersions. Although (6, 5) chirality SWCNTs are semiconducting, at the 3 kHz frequency used in this work, both metallic and semiconducting SWCNTs are much more conductive than the surrounding media and are thus similarly polarizable.^100,101^ We further note that, in fluid suspension, individual SWCNTs have been characterized as having persistence lengths on the order of a few to tens of microns.^102–104^ At these persistence lengths, SWCNTs on the order of 1 μm behave as rigid rods. Nevertheless, if particles were nonrigid or otherwise have curvature, then the optical-polarimetry length measurement technique would probe the distance between the endpoints.

The optical-polarimetry and AUC distributions shown in Figure 3 show consistency in the peak locations, with both showing peaks around 1.5 μm for the longer sediment fraction, and 1.0 μm for the shorter supernatant fraction. The two techniques also agree in showing the expected trend that the SWCNTs in the sediment fraction in Figure 3(a) are indeed longer than those of the supernatant in Figure 3(b). Some differences are notable, with optical polarimetry being less sensitive to submicron SWCNTs than AUC, whereas the directly interpreted AUC measurements can overpredict an extended tail of the distribution toward longer SWCNT lengths.^105^ The key observation in Figure 3 is the clearly differentiated mass-weighted mode lengths for the two fractions, which are independently distinguished by both techniques and are in good agreement. AUC provides a good comparison data set because both techniques measure the length distribution in free dispersion, without interactions with a surface, and with similar weighting as both use absorbance signals as their input data.

**Figure 3:**
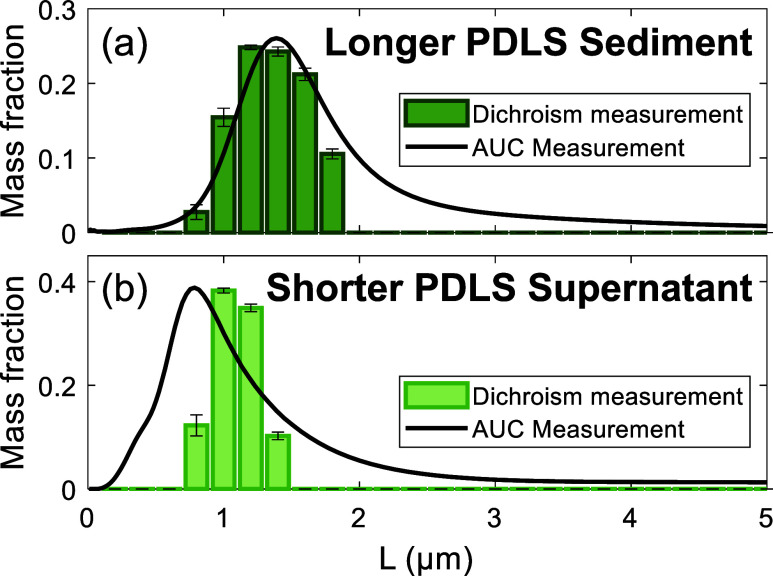
Validation of the technique by comparison to AUC measurements in (a) sediment and (b) supernatant fractions of PDLS-separated CoMoCAT SWCNTs. For both fractions, the dichroism-derived length histograms show relatively narrow distributions centered around ≈1.5 μm in the sediment and ≈1.0 μm in the supernatant after PDLS. The AUC measurements yield broader distributions with a longer tail extending to several microns in length, which may be due to particle–particle interaction and agglomeration in the samples.

### Sensitivity to Diameter

It is desirable for the *E*-field-assisted dichroism length-measurement technique to be insensitive to particle diameter since the diameter is assumed and may be poorly known or widely varying in a given sample. Fortunately, the *E*-field particle alignment only depends weakly on diameter as the inverse log of aspect ratio α = *L*/*d* and depends more strongly on particle length, explicitly scaling as *L* ^3^/(ln 2α – 1), as described in the Supporting Information. As an example, Figure 4 shows the length distributions extracted from a measured dichroism signal assuming two different diameters: 100 (the size of narrow bundles, as observed in SEM) and 263 nm (representing the average diameter for the bundled SWCNTs as observed in SEM). Despite the over 2-fold difference in assumed diameters, the extracted mean length only differs by 11%, which demonstrates the robustness of the method to variations in particle diameters.

**Figure 4:**
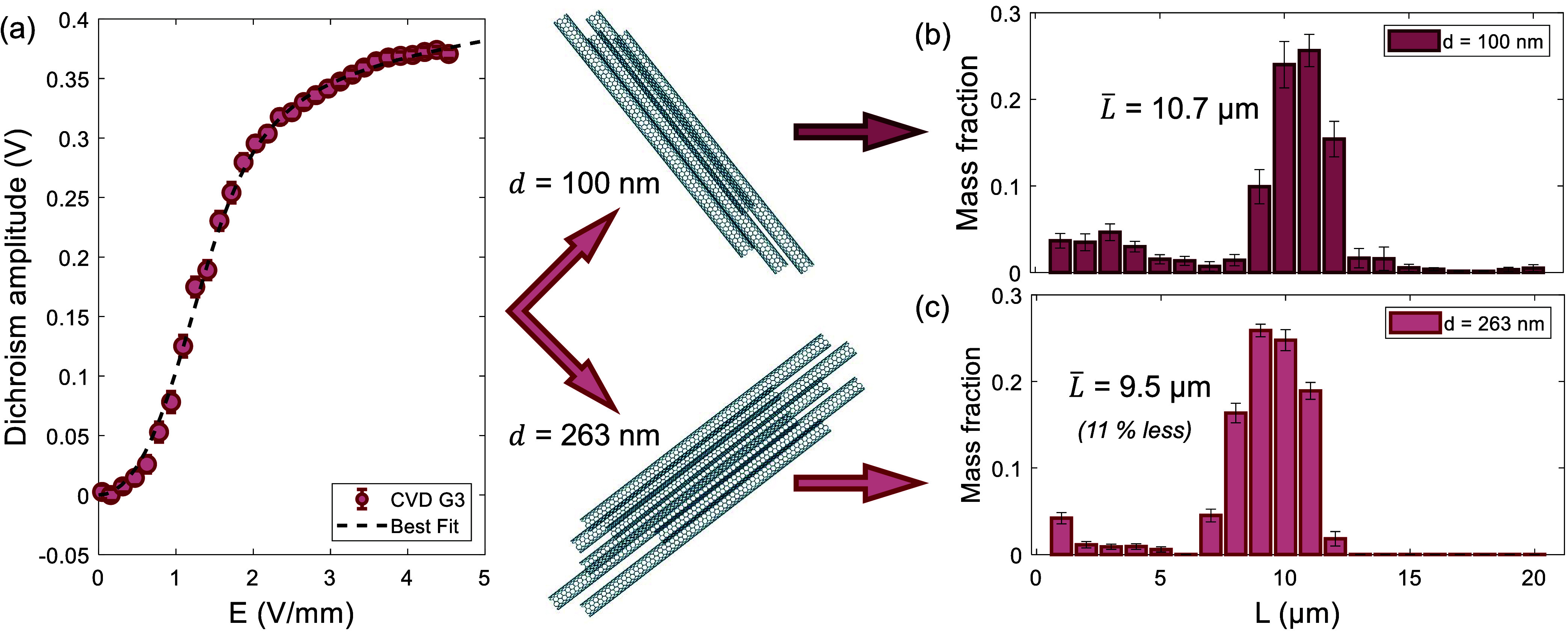
Diameter dependence of the extracted length. Programmatically, the diameter is an assumed input parameter, and this figure probes the effects of different assumptions for diameter on the same data set. (a) Dichroism response amplitude. (b) With an assumed particle diameter of 100 nm, the extracted lengths have a mean of 10.7 μm. (c) When the assumed particle diameter is 263 nm, the length distribution shifts its mean to 9.5 μm, only an 11% reduction, demonstrating that the measurement is insensitive to particle diameter. Parts of figure were created with BioRender.com.

### Effect of Sonication on Sub-Nanometer-Diameter SWCNTs

Having demonstrated consistency of the optical-polarimetry technique against SEM, optical microscopy, and AUC on Ag nanowires, wafer-grown SWCNT forests, and PDLS-fractionated CoMoCAT SWCNTs, we next studied the change in length distribution of sub-nanometer-diameter SWCNTs and SWCNT bundles with bath and tip sonication of different durations and powers. These SWCNTs and SWCNT bundles were obtained from CHASM Advanced Materials, Inc. While the SWCNTs were 0.8 nm in diameter, measured by optical fluorescence,^106^ the SWCNT bundles had lengths *L* ≤ 7 μm and diameters of 5.1 nm, as determined by SEM and TEM imaging. These ultranarrow SWCNTs are difficult to measure by other methods, as their small diameter limits SEM and optical imaging, and their extreme aspect ratio makes statistical AFM or TEM length measurements tedious and impractical. The optical-polarimetry method does not differentiate between individual SWCNTs and SWCNT bundles but instead reports the mass-weighted length distribution of particles in solution.

For the 0.8 nm SWCNTs and SWCNT bundles, the effect of higher-power tip sonication at 180 W for 180 s was compared to that of two different durations of milder-power bath sonication, 18 h and 45 min. Concentrations of 0.1 mg/L were used for bath-sonicated samples, while the tip-sonicated sample was further diluted to 0.01 mg/L before sonication to further reduce the particle–particle interaction. In spite of the lower concentration, the dichroism amplitude saturation levels observed in Figure 5(a) for the tip-sonicated sample have a higher magnitude, indicating a greater number of particles in suspension. The resulting length distribution seen in Figure 5(b) shows that when tip sonication is applied, the 0.8 nm diameter SWCNTs in DCB shorten significantly, with 77% of the mass of SWCNTs (as attributed by optical absorbance signal) breaking into shorter segments ≤1.2 μm. The remaining SWCNTs have lengths ranging from 2 to 5 μm, resulting in a bimodal distribution. For SWCNT samples that underwent milder bath sonication for 18 h, the population of SWCNTs 2 to 5 μm long is much higher and only shows one peak, as seen in Figure 5(c). A decreased duration (45 min) of bath sonication preserved the length of the SWCNTs and narrowed the distribution, as shown in Figure 5(d). The longer tail seen in Figure 5(c) is believed to be due to the extended bath sonication breaking large agglomerates into long bundles in the range of 3.5 to 6 μm. For shorter bath sonication times, the larger agglomerates that remained either had small aspect ratios or settled out of solution and thus were mostly not detected. The SEM image shown in the inset of Figure 5(a) further demonstrates that thick SWCNT bundles existed in suspension at the measured length range on the order of several microns. However, SEM imaging cannot be used for a direct statistical comparison to the optical-polarimetry measurement since it does not resolve individual sub-nanometer-diameter SWCNTs. In comparison, the optical-dichroism technique is able to measure not only bundles but individualized sub-nanometer-diameter SWCNTs.

**Figure 5:**
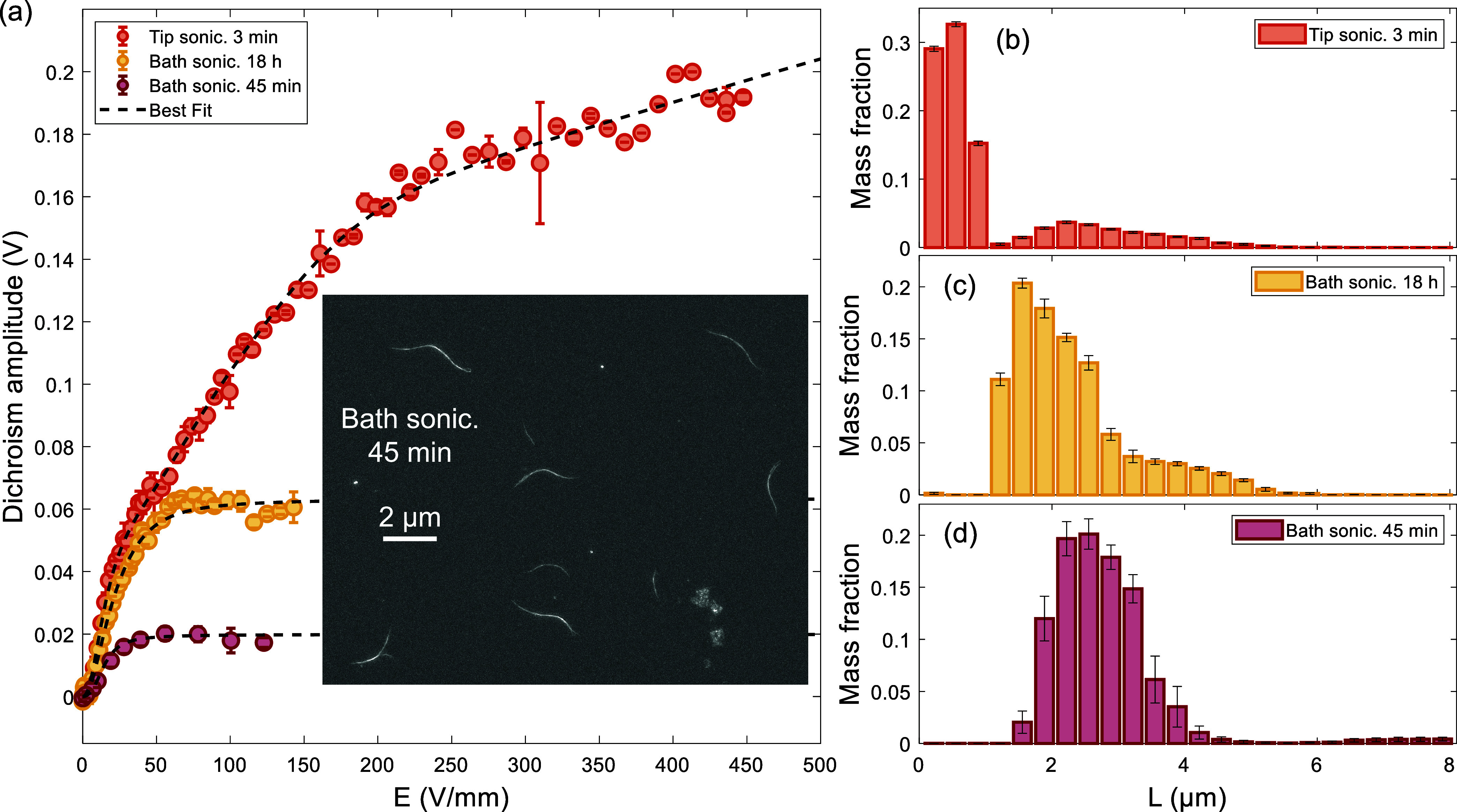
Variation of CoMoCAT SWCNT length distributions with different sonication types and durations. (a) Dichroism signal response for CoMoCAT SWCNTs for three sonication conditions. Inset: SEM image of the SWCNTs treated with 45 min of bath sonication, where ≈2 to 3 μm SWCNT bundles are visible. (b) Dichroism-measured length distribution for SWCNTs treated with high-power tip sonication. Error bars show the standard deviation from 10 best fits of the dichroism data, starting from random initial conditions. The high mass fraction of shorter ≤1.2 μm SWCNTs shows that tip sonication fractured many of the CNTs. (c) Extended duration of bath sonication increased the mode to 2 μm, and showed a greater content of longer ≈4 μm CNTs that were dispersed from agglomerates. (d) Shorter duration of bath sonication yielded the longest SWCNTs, with a length distribution centered around 3 μm.

### Range of Applicability of Optical-Polarimetry Length Measurement

The optical-polarimetry length measurement method is complementary to other measurement methods for different samples, offering a fast, cheap, and relatively simple method that covers the gap in other measurement techniques. It is best suited for 1D nanoparticles in the 0.5 to 15 μm length range that show optical dichroism and are electrically polarizable in an insulating solvent like hexadecane or DCB. If a high-aspect-ratio particle exceeds 15 μm in length, the dichroism method can begin to lose fidelity due to a low optical absorption signal at concentrations low enough to be stable in applied *E*-fields. For such very long samples, direct SEM or optical microscopy, although tedious because it requires a deposition and drying step, may produce the most robust results. Additionally, laser scattering or laser diffraction may also provide qualitative measurements on long 1D nanoparticles.^37,40,41^ On the lower bound, particles below 0.5 μm require high *E*-fields to achieve a high degree of alignment (>500 V/mm to hit *S* = 0.95), and although the dielectric strength of pure solvents can extend into the hundreds of kV/mm,^107^ impurities in the solvent, impurities from the sample, or the particles themselves can limit the voltage stability of the solution. Such constraints define the lower bound of the measurable length range. In comparison, AUC and AFM are better suited for measuring shorter nanoparticles in the submicron range, and are less useful for several-micron-long particles.^66^ AFM and AUC have the important advantage of not being restricted to polarizable particles that show optical dichroism. However, longer particles are tedious to measure using AFM, and measurements of long or dense particles by AUC suffer from inaccuracies due to assumed hydrodynamic scalings or fast sedimentation on the order of the time it takes for the AUC to reach steady state. Within its range of applicability (polarizable 1D nanoparticles in the 0.5 to 15 μm length range, stable in dilute suspension in low-conductivity solvents), the *E*-field-assisted optical-polarimetry method is a particularly efficient and accurate method to measure the length distribution of 1D nanoparticles in suspension.

## Conclusion

Knowledge of the length distribution and dispersion state of 1D nanoparticles is critical to accessing their unique properties in many applications. Here, we have demonstrated an efficient, *E*-field-assisted optical technique to measure the length distribution of high-aspect-ratio particles in a suspension. The technique addresses measurement of particles that are otherwise difficult to characterize, and is applicable to any electrically polarizable 1D particle that displays linear dichroism, including sub-nanometer-diameter carbon nanotubes and metal nanowires. It is also inherently compatible with liquid-phase processing. The method can efficiently quantify the statistics of the length of 1D nanoparticles in dilute suspension over a range of approximately 0.5 to 15 μm, with the upper limit set primarily by the stability of the particles in suspension. As the *E*-field alignment depends strongly on particle length but only weakly on aspect ratio, the measurement has the benefit of being relatively insensitive to diameter, which may vary within a sample or be poorly known. We anticipate that this new method, by making possible the efficient statistical characterization of the length distribution of polarizable 1D nanoparticles in solution, may enhance our fundamental understanding of the properties and processing of 1D nanomaterials and accelerate the development of their technological and commercial applications.

## Methods

Certain equipment, instruments, software, or materials, commercial or noncommercial, are identified in this paper in order to specify the experimental procedure adequately. Such identification is not intended to imply recommendation or endorsement of any product or service by NIST, nor is it intended to imply that the materials or equipment identified is necessarily the best available for the purpose.

### Materials

Silver nanowires PL-AgW100 from PlasmaChem GmbH were used as received. CoMoCAT SWCNTs were obtained from Sigma-Aldrich, product no. 704148. Hexadecane was purchased from Fisher Scientific, product AC120465000. Dichlorobenzene was purchased from Sigma-Aldrich, product number 240664. SWCNTs acquired from CHASM Advanced Materials are listed under product number CG400 lot PXD2–2024.

### Optical Dichroism Measurement

For optical dichroism measurement, a He–Ne laser was used to produce 632.8 nm laser light at 15 mW (Melles Griot, 6628002–04), which was then passed through a polarizer with a 10^–5^ extinction ratio (Thor Laboratories, GT5). Following this, the laser beam passed through a rotating half-wave plate (Thor Laboratories, WPHSM05–633), which was rotating between 20 and 25 Hz. The laser was then incident on the sample, passing through a 4 cm optical glass cuvette (Starna Cells, 1-SOG-40) holding the sample and two 316L stainless steel electrodes for the application of the *E*-field. A voltage was applied to the CNT sample through the parallel-plate electrodes by connecting a function generator (Tektronix Inc., model AFG1062) to a high-voltage amplifier (TREK, Inc., model PZD350 M/S, or TREK, Inc., model 609E-6). Defined voltage pulses were triggered by a timing box (BNC Precision, model 555). After the sample cell, the laser finally struck the photodiode detector (Thor Laboratories, DET210). Voltages were acquired using a National Instruments DSA-4472 digital lock-in amplifier, which simultaneously recorded the optical signal from the photodiode and the signal from the motor encoder.

### Theory and Fitting Algorithm

The method used for fitting the dichroism signal is designed to be highly robust as the measured signal represents a superposition of contributions from particles of different lengths. The length distribution of 1D nanoparticles was determined by fitting the field-dependent dichroism to a theoretical model describing field-induced alignment. For each trial particle length, the alignment order parameter was calculated using the Maxwell–Wagner polarization model^76^ combined with the effects of Brownian motion. The overall dichroism response was then expressed as a weighted sum of contributions from particles of different lengths, with each weight corresponding to the mass fraction of particles of that length.

To converge on the fit, the coefficients were randomly initialized and iteratively adjusted to minimize the fitting error, with each cycle recalculating the model response and applying progressively smaller updates through 35 adjustment cycles, utilizing the principle of relaxation to impart convergence stability. To evaluate the robustness and uniqueness of the solution, the entire fitting procedure was repeated 10 times with randomized initial conditions, and the experimental data points were each resampled according to the standard deviation of the data points. The standard deviations of the results of these solutions are shown as the error bars in each length histogram depicted in this work. Videos illustrating the fitting algorithm are provided in the Supporting Information, showing the refinement process and how these multiple repetitions are performed to generate independent length distributions with different starting conditions. An explicit description of the fitting algorithm is provided in the Supporting Information.

### CVD Growth of SWCNT Forests

For CVD growths G1 to G3 used for measurement validation, SWCNTs were CVD-grown as aligned forests of uniform length using a Fe/Mo catalyst (5.5/0.5 Å) on a 100 mm Si wafer in an AIXTRON Black Magic reactor from an acetylene feedstock, as described elsewhere.^89–91^ More details about the CVD-grown SWCNTs can be found in the Supporting Information.

### CoMoCAT Length Sorting

Two length fractions of CoMoCAT-type SWCNT material were isolated using PDLS^98^ from a previously dispersed, purified, and length-enriched population generated in the production of NIST RM 8281. In brief, SG65-grade CoMoCAT SWCNT material was dispersed *via* tip sonication into 20 g/L sodium deoxycholate (DOC) solution, followed by centrifugation to remove poorly dispersed material, and a subsequent ultracentrifugation-based length fractionation *via* an upward race through a dense liquid medium.^61^ The specific fraction used as the parent for this effort was F11, a fraction containing longer SWCNTs than incorporated in the long fraction of RM 8281.^108^ For PDLS, poly­(methacrylic acid) (PMAA) solution in water was added to an aliquot of F11 SWCNT dispersion to a concentration of 10 g/L. The suspension was allowed to rest for 24 h and then ultracentrifuged at 1811 rad/s in an SW32.1 rotor for 20 min. Following this, the supernatant (fraction S1) was decanted, and the sediment was retained. To improve fractionation fidelity, the separated supernatant (still containing 10 g/L PMAA) was rested for an additional 24 h, and the ultracentrifugation repeated, again decanting the supernatant and retaining the sediment. The supernatant was then subjected to repeated ultrafiltration against a 100 kDa membrane and back dilution with fresh surfactant solution to remove the PMAA polymer and concentrate the SWCNTs into a 10 g/L DOC environment; this sample, measured as described above, is referred to as “supernatant.” Both sediments were resuspended with 10 g/L of DOC solution, combined, and then processed with another pass of PDLS. For this pass, 6 g/L PMAA was used, but otherwise the procedure was equivalent. The resulting supernatant (fraction P1S0.6) was decanted, subjected to ultrafiltration to remove the PMAA polymer and concentrate the SWCNTs into a 10 g/L DOC environment, and then analyzed as described above, referred to as “sediment.”

### AUC Measurements

The AUC data were collected using a Beckman-Coulter XL-I analytical ultracentrifuge^66,109^ in sedimentation velocity mode at 2932 rad/s (28 krpm) in an AN-50 rotor at 20 °C (>2 h preequilibration) and an absorbance wavelength of 345 nm. Sedimentation coefficient distributions were determined using SEDFIT v17, using the *c*(*s*) model and previously determined solution and nanotube parameters, and converted to length distributions.^98,109^

### Imaging

To prepare CNT samples for SEM images, as shown in Figures 2 and 5, CNTs were sonicated in 1,2-dichloroethane (DCE) and then dried on a Si wafer. DCE was chosen as a solvent because it is volatile, dries quickly to leave evenly distributed CNT bundles on the Si wafer, and has a similar solvency for CNTs compared to DCB. Samples of Ag nanowires were sonicated in ethanol because it is a volatile solvent which disperses the nanowires well. For optical images used to generate length distributions, a drop of the same samples that were used for the dichroism measurement was placed on a glass slide and covered with a coverslip, then allowed to settle for 10 min. The Ag nanowires or CNT bundles deposited on the slides were then observed under bright-field illumination with a 40× objective on an Olympus IX71 microscope.

## Supplementary Material









## Notes

Additional details on the optical-polarimetry measurement setup; theoretical modeling of *E*-field polarization of conducting particles and resulting laser dichroism; fitting algorithm to fit the theoretical model of a length distribution with measured optical laser dichroism; discussion on algorithm results as a function of the number of bins; description and images of CVD growth of SWCNT forests on a 100 mm Si wafer; and description of the method to transfer SWCNTs in aqueous sodium deoxycholate to a surfactant-free organic DCB phase (PDF)
Videos of the fitting algorithm converging on a fit solution, employing a varying number of bins, described further in Figure S4:
Fitting video—Tip sonic. 3 min Figure 5(b)—12 bins (Video S1) (MP4)
Fitting video—Tip sonic. 3 min Figure 5(b)—24 bins (Video S2) (MP4)
Fitting video—Tip sonic. 3 min Figure 5(b)—47 bins (Video S3) (MP4)
