# Supplementary material for: Efficient Measurement of Length Distribution of 1D Nanoparticles in Solution via Optical Polarimetry
Source: ACS Nano. 2025 Dec 1;19(49):41765–76. doi: 10.1021/acsnano.5c14994 (PMC12713781; doi:10.1021/acsnano.5c14994)
Supplement: Supplementary file 4 [file nn5c14994_si_004.pdf]

Supporting Information

Efficient measurement of length distribution of 1D nanoparticles in solution *via* optical polarimetry

Richard J. Castellano,*,†,‡ Da-Chi Yang,† Sei Jin Park,¶ Pavel Shapturenka,§ Robert F. Praino,‡ Jeffrey A. Fagan,§ Francesco Fornasiero,¶ and Jerry W. Shan†

†*Department of Mechanical & Aerospace Engineering, Rutgers, The State University of New Jersey, New Brunswick, NJ 08854, USA*

‡*CHASM Advanced Materials, Inc., Canton, MA 02021, USA*

¶*Physical and Life Sciences Directorate, Lawrence Livermore National Laboratory, Livermore, CA 94550, USA*

§*Materials Science and Engineering Division, National Institute of Standards and Technology Gaithersburg, MD 20899, USA*

E-mail: rick.castellano@rutgers.edu

Product Disclaimer

Certain equipment, instruments, software, or materials, commercial or non-commercial, are identified in this paper in order to specify the experimental procedure adequately. Such identification is not intended to imply recommendation or endorsement of any product or service by the National Institute of Standards and Technology (NIST), nor is it intended to

imply that the materials or equipment identified are necessarily the best available for the purpose.

Optical Polarimetery Measurement Setup

The laser setup can be seen in Fig. S1(a), depicting the optics used for the dichroism measurement. The laser was allowed to warm up for 30 min after being energized such that the laser intensity had stabilized. The first optics are a confocal diverging (focal distance f_d = 25 mm) and converging lens (f_d = 40 mm) pair intended to broaden the laser beam, widening the spot, and making the intensity more uniform. An aperture was next on the laser path, and set such that the laser beam was not incident on the polished stainless steel electrodes, as reflections could be partially polarized. A subsequent Glan-Taylor polarizer with a 10^{-5} extinction ratio ensured that the laser was highly polarized before passing to the half-wave plate. After the wave plate, the laser passes through the sample in a cuvette, before finally striking the photodiode, similar to previous work.[1] Also, due to the fact that the He-Ne laser (632.8 nm) was initially polarized, rotations of the polarizer could be used to modulate the incident laser intensity. This was performed to achieve a photodiode voltage between 6 V and 8 V for the best signal-to-noise ratio, seen as a red sine-wave in Fig. S1(b). The rotating half-wave plate was driven by a motor (Faulhaber, model 3557F0052) with an attached encoder (US Digital, item MAE3-A10-250-500-7-B) which was connected to the lock-in amplifier, seen as the blue sawtooth wave in Fig. S1(b). The red sine-wave is seen to oscillate at four times the frequency of the blue sawtooth wave, which is due to the fact that carbon nanotubes (CNTs) absorb polarized light with 180° symmetry and that the angle of polarization is twice the angle of the half-wave plate. To ensure that samples were stable over the course of the measurements, samples were examined at least twice, with a 5 min delay between scans. Only data with consistent measurements for both scans are reported in this work, and if scans demonstrated hysteresis, the samples were diluted, sonticated and measured again. The polished 316L stainless steel electrodes immersed in dichlorobenzene

(DCB) solution and polytetrafluoroethylene (PTFE) spacers were custom-made and can be seen in Fig. S2.

This work has many similarities to other works involving E-field alignment of particles and optical polarimetery to examine the degree of alignment of particles. Work on the polarizability of CNTs as measured by optical polarizability[2] measures the alignment order parameter, but does not comment on the lengths of particles. A similar dichroic measurement method is presented[1] and makes interesting comments on the ratio of electric to shear-flow torques, but does not measure the length of particles. Even others use E-fields to align particles, then examine Brownian relaxation timescales to extract length distributions;[3,4] birefringence measurements of the particle alignment relaxations are presented with a method of extracting length distribution of the particles. Extraction of length from the thermal relaxation time by mobility-based calculations relies on modeling of the rotational diffusion of particles which can be sensitive for particles of exceedingly low diameter, especially if surfactants are employed.

(a) (b)

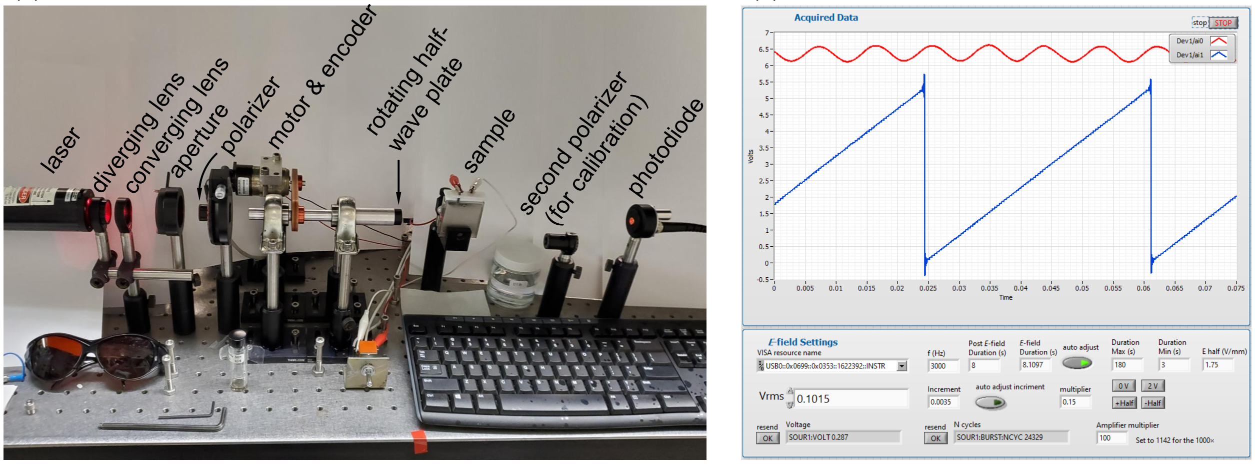

Figure S1: The optical polarimetery equipment. (a) optical setup. (b) acquired data with LabVIEW front panel visible.

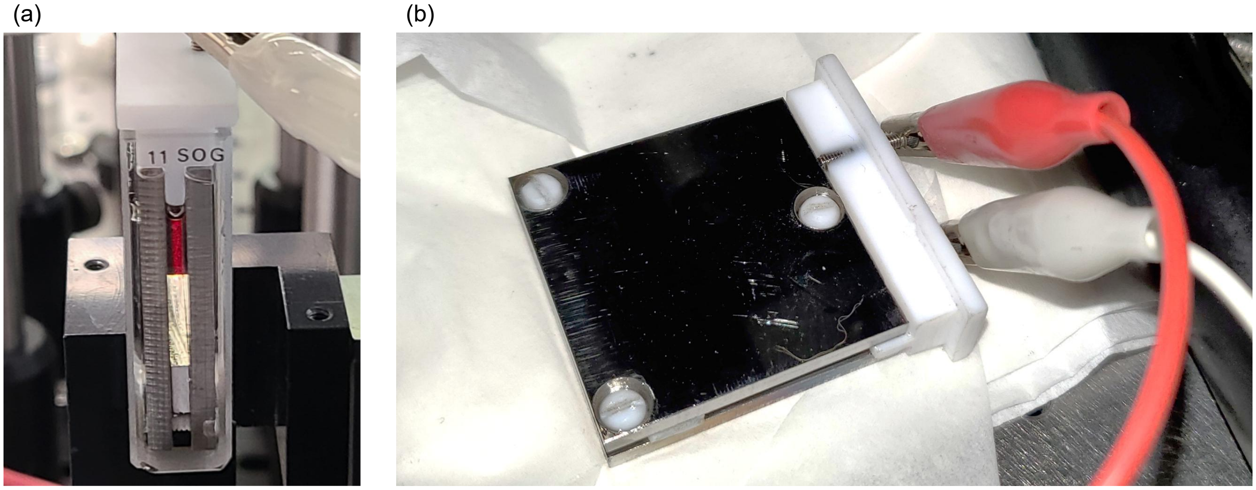

Figure S2: Images of the cell and electrodes. (a) Image of the 4 cm cuvette with stainless steel 316L electrodes immersed. The laser passing through the dispersion sample is visible due to the optical scattering of the laser light. (b) Image of the stainless steel electrodes and PTFE spacers and screws to maintain a uniform 2.24 mm gap. Electrode wires are electrically connected to the parallel plates with screws.

Theory

The Maxwell-Wagner polarization model predicts that if the particle has either a different electrical conductivity or permittivity than that of the fluid, it will become electrically polarized when subjected to an electric field (E-field) in fluid suspension. This model can be used to quantify the maximum orientational potential energy as the potential energy difference between a particle aligned perpendicular, from one aligned parallel to the E-field. This alignment potential energy U is highly dependent on the particle length L, and only slightly influenced by the particle diameter d. For ellipsoidal particles, the theoretical aligning potential energy U_{MW} has an analytical solution from the Maxwell-Wagner polarization model[5] defined as a function of the complex permittivity of the particle and fluid $\underline{\varepsilon}_{p,f} = \varepsilon_{f,p} + \sigma_{f,p}/i\omega$, where $\varepsilon_{p,f}$ is the permittivity of the particle or the suspending fluid, $\sigma_{p,f}$ is the electrical conductivity of the particle or the suspending fluid, ω is the angular frequency of the E-field, and i is the imaginary unit:

$$U_{\mathrm{MW}} = \frac{1}{2}\varepsilon_f V_0 E_{rms}^2 Re\left\{\left(\left(\frac{\underline{\varepsilon}_p}{\underline{\varepsilon}_f} - 1\right)^{-1} + L_{\parallel}\right)^{-1} - \left(\left(\frac{\underline{\varepsilon}_p}{\underline{\varepsilon}_f} - 1\right)^{-1} + L_{\perp}\right)^{-1}\right\}. \quad \text{(S1)}$$

Here, V_0 is the particle volume, and $L_{\parallel,\perp}$ are the depolarization factors along the principal axis of the particle and perpendicular to the principal axis, respectively, and $Re\{\ \}$ is the real part of the complex argument, and $L_{\perp} = \frac{1}{2}\left(1 - L_{\parallel}\right)$. For prolate ellipsoids,[5]

$$L_{\parallel} = \alpha^{-2}\left(-e_{cc}^{-2} + \frac{1}{2e_{cc}^3}\ln\frac{1+e_{cc}}{1-e_{cc}}\right), \quad \text{(S2)}$$

where $e_{cc} = \sqrt{1-\alpha^{-2}}$ is the eccentricity of the ellipsoid. In the limit of $\alpha \gg 1$, *i.e.*, large aspect ratio, $L_{\parallel} \approx \alpha^{-2}(\ln 2\alpha - 1)$ and $L_{\perp} \approx 1/2$. With the further assumption that CNTs are much more conductive than the suspending fluid, $\|\underline{\varepsilon}_p\| \gg \|\underline{\varepsilon}_f\|$, this simplifies to

$$U_{\mathrm{MW}} = \frac{\pi\varepsilon_f L^3 E_{rms}^2}{12(\ln 2\alpha - 1)}. \quad \text{(S3)}$$

A small correction factor can be applied for cylindrical particles[6] as

$$U_{\mathrm{MW}} = \frac{\pi\varepsilon_f L^3 E_{rms}^2}{12(\ln 2\alpha - 1)}\left(1 - \frac{4/3 - \ln 2}{\ln 2\alpha - 1}\right). \quad \text{(S4)}$$

The exact transition to where a particle can be considered highly conductive is given by $\left\|\frac{\underline{\varepsilon}_p}{\underline{\varepsilon}_f}\right\| \frac{\ln 2\alpha - 1}{\alpha^2} \gg 1$. For instance, according to Eq. S1 if $\left\|\frac{\underline{\varepsilon}_p}{\underline{\varepsilon}_f}\right\| \frac{\ln 2\alpha - 1}{\alpha^2} = 10$, then U_{MW} would be 91 % of its maximal value for a perfectly conductive particle, theoretically resulting in only a 3.1 % difference in measured length.

The ratio of the alignment potential energy to the thermal energy, $k_B T$, directly controls the probability distribution of the degree of alignment of the 1D particles in suspension. Here, k_B is the Boltzmann constant 1.38×10^{-23} J/K, and T is the absolute temperature. This results in a Boltzmann distribution

$$P(\theta) \propto \exp\left(-\frac{U}{k_B T}\sin^2\theta\right)\sin\theta, \tag{S5}$$

or without loss of generality if θ is constrained to $[0, \pi/2]$,

$$P(\theta) = \frac{\sqrt{\frac{U}{k_B T}}}{D\left(\sqrt{\frac{U}{k_B T}}\right)}\exp\left(-\frac{U}{k_B T}\sin^2\theta\right)\sin\theta, \tag{S6}$$

where $D(x)$ is Dawson's integral.

The dichroic absorption of suspensions of thin rods is taken to be directly proportional to the alignment-order parameter,[7–11] which is given as $S = \frac{1}{2}\langle 3\cos^2\theta - 1\rangle$, where $\langle\ \rangle$ represents the ensemble average; explicitly,

$$S = \frac{1}{2}\int_0^{\pi/2} P(\theta)(3\cos^2\theta - 1)d\theta. \tag{S7}$$

Therefore, at each E-field strength, every tube length will produce a unique alignment order parameter, as seen in Fig. S3(a). The curves seen in Fig. S3(a) show three distinct regimes: in Regime 1 the particles experience a low degree of alignment, in Regime 2 the particles experience a higher degree of alignment as the E-field strength increases, and in Regime 3 the particles are fully aligned and do not increase in degree of alignment as the E-field strength increases further. These three regimes are seen again in Fig. S3(b) as a function of the particle length and E-field strength. In order to fully capture the dichroic response of any given particle, E-field strengths should be chosen to cover all three regimes. The dichroism amplitude of the measured optical signal is directly proportional to S, and if E-field strengths are chosen to range high enough to observe the saturation of the alignment order parameter, then the relationship between S and the dichroism amplitude can be quantified.

The permittivity of the solvents used were taken from the literature[12] to be $\varepsilon_f = 10.12\varepsilon_0$ for DCB and $\varepsilon_f = 2.046\varepsilon_0$ for hexadecane, where $\varepsilon_0 = 8.85 \times 10^{-12}$ F/m is the permittivity of free space.

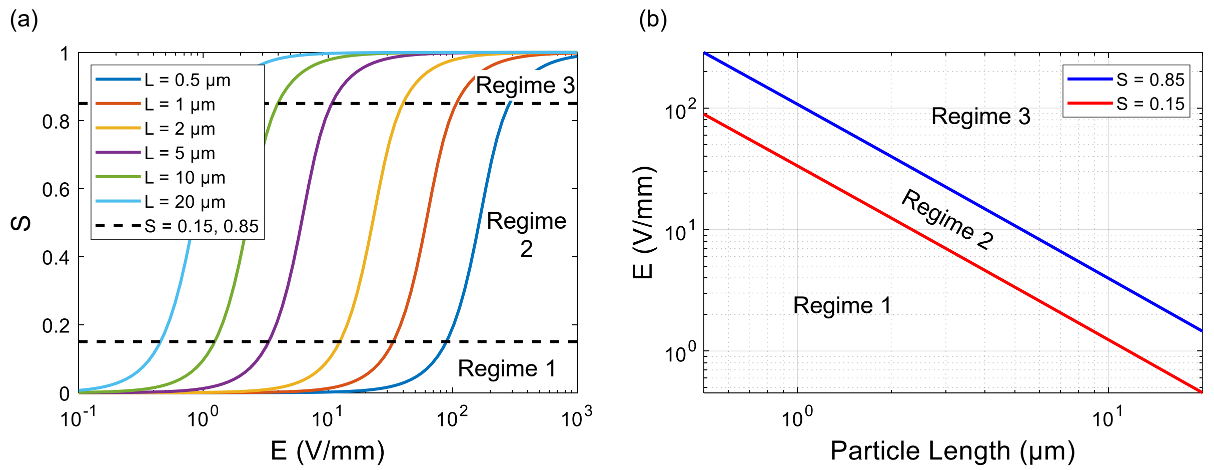

Figure S3: Regimes of dichroism response. (a) Alignment order parameter S as a function of E. Longer particles will experience a high degree of alignment at lower E-fields. (b) Map of the three alignment regimes as a function of the particle length and E-field. To accurately measure particles of a given length, E-fields must be chosen to cross through all three regimes for that length.

Fitting

To find the length distribution of 1D nanoparticles, we first calculate the theoretical alignment order parameter for polarizable rods of a given length, taking a Boltzmann distribution for the nanoparticle orientation under the combined influence of E-field alignment and Brownian motion. Each particle length L_i will produce a unique alignment-order parameter $S_i(E)$ at every field strength E, as can be seen in Fig. S3(a) and is described in Eqs. (S4) and (S6). Assuming the dichroic absorption of the suspension of 1D polarizable particles to be directly proportional to the alignment-order parameter,[2,7,9] we calculate the resulting dichroism amplitude as a linear combination of $S_i(E)$ for each particle lengths L_i as

$$A_{\text{model}}(E) = \sum c_i S_i(E). \tag{S8}$$

To find the length distribution of particles, we adjust the weights c_i to best fit the calculated $A_{\text{model}}(E)$ to the measured absorption anisotropy. The mass fraction can then be converted to a number fraction by dividing by L_i for each length. To fit the dichroism amplitude A

as a function of E, trial lengths L_i were chosen with the criteria that the alignment order parameter as a function of E covers both low S and high S values for the experimentally applied E-field strengths. The linear combination coefficients c_i defined in Eq. (S8) are interpreted as the mass fraction[13,14] of each contributory length as $w_i = c_i / \sum c_j$, where w_i is the mass fraction of CNTs of length L_i.

Due to the large number of parameters used in the fitting process, and to address potential issues arising from the non-uniqueness of the solution, the fitting algorithm was run 10 times using randomized initial conditions to comment on the degree of certainty of the extracted distribution, as described below:

1. The measured dichroism data at each E-field was treated as a random normal variable. For each fitting iteration, the dichroism data was sampled according to the mean and standard deviation of the measured data.

2. An initial guess was generated for each combination coefficient c_i as a random number varying in the interval $(0, 2A_{max}/N)$ where A_{max} is the maximum of the measured dichroism, and N is the number of trial lengths.

3. An initial value for Δc was taken as A_{max}/N.

4. The coefficients c_i were placed in a random permutation.

5. Starting with the first coefficient in the permutation, each coefficient was varied by attempting 7 trial values evenly spaced from $c_i - \Delta c$ to $c_i + \Delta c$, and c_i^* the positive coefficient that produced the minimum least-squared error between the theoretical dichroism defined in Eq. (S8) and the measured data was selected.

6. The value of c_i was replaced by a relaxation step towards the new found minimum as $c_i \leftarrow 0.6c_i^* + 0.4c_i$.

7. After all coefficients were iterated upon with steps 5 and 6, the value of Δc was reduced to 80 % of its prior value.

8. Steps 4 to 7 were repeated 35 times, and the final values of the coefficients produced a length-distribution histogram.

9. Steps 1 to 8 were repeated 10 times, and the resulting 10 histograms allowed for the calculation of means and standard deviations for each bar in the histogram.

This method is robust with respect to the number of bins used in constructing the length distribution. As shown in Fig. S4, very similar distributions are obtained when 12, 24, and 47 bins are chosen. While increasing the number of bins improves the resolution of the resulting histogram, it does not significantly alter the overall shape or accuracy of the extracted distribution. This indicates that the method reliably captures the length profile of the sample independent of bin selection. Videos of the fitting algorithm which produced Fig. S4(a), (b), and (c) can be found in the Supporting Information.

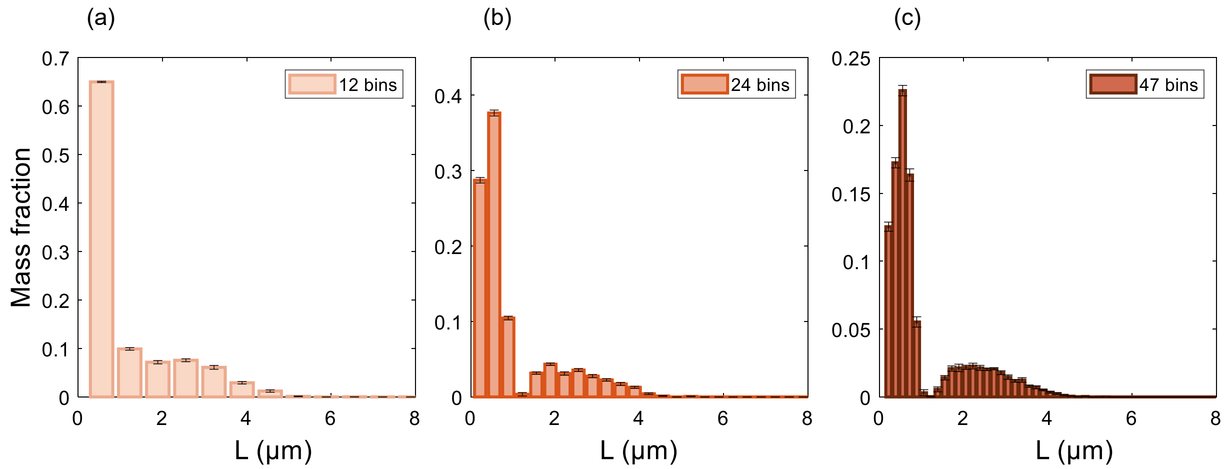

Figure S4: The effect of choosing a different number of bins for CoMoCAT SWCNTs treated with 3 minutes of tip sonication. See Supporting Information for videos of the fitting algorithm converging on a solution. (a) At 12 bins there is insufficient resolution to observe a bimodal distribution. (b) At 24 bins a distinct bimodal distribution is visible. (c) With 47 bins, the distribution is not different from 24 bins, demonstrating the insensitivity of the fitting algorithm to bin resolution.

CVD Growth of SWCNT Forests

Single-walled carbon nanotubes (SWCNTs) for samples G1 to G3 were CVD-grown as aligned forests of uniform lengths using a 5.5/0.5 Å Fe/Mo catalyst on a 100 mm Si wafer in an AIXTRON Black Magic reactor with acetylene feedstock, at Lawrence Livermore National Laboratory (LLNL), as detailed in the literature.[15–17] These wafers, with (1.2 to 2.4) $\times$ 10^{12} CNTs/cm^2 can be seen in Fig. S5(a), where the forests were scraped to reveal the Si wafer, and the uniform-length SWCNTs. These wafers were sliced into 1 cm^2 squares, and placed into DCB, then sonicated in a bath to gently detach the CNTs as bundles while preserving their length. Images of such CVD-grown bundles can be seen after drying from their dispersed state on a Si wafer under SEM in Fig. S5(b), measurements from which were used for comparison. For length measurement in the optical-polarimetery system, the SWCNT bundle dispersion was diluted to 0.5 mg/l and then measured.

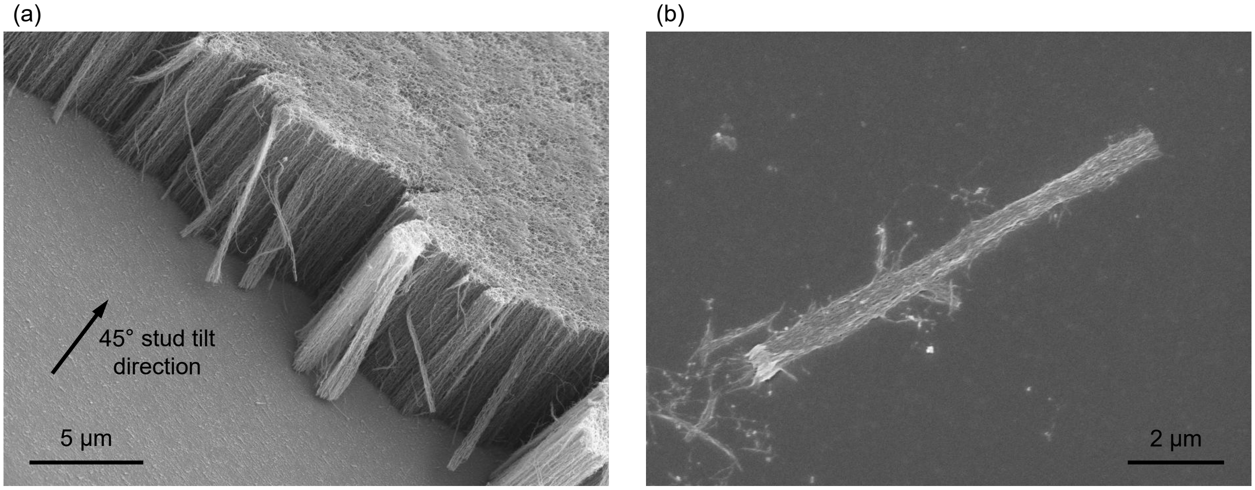

Figure S5: SEM images of CVD SWCNTs. (a) Image of the SWCNT forest grown on a Si wafer. The CNT forest was scratched to reveal the Si wafer and the grown forest of aligned SWCNTs, and placed on a 45° stud to visualize the vertically aligned CNT forests, which in this image are 10.3 µm long. (b) Image of a CVD SWCNT bundle $\approx$ 1 µm in diameter and 9.5 µm in length from G2.

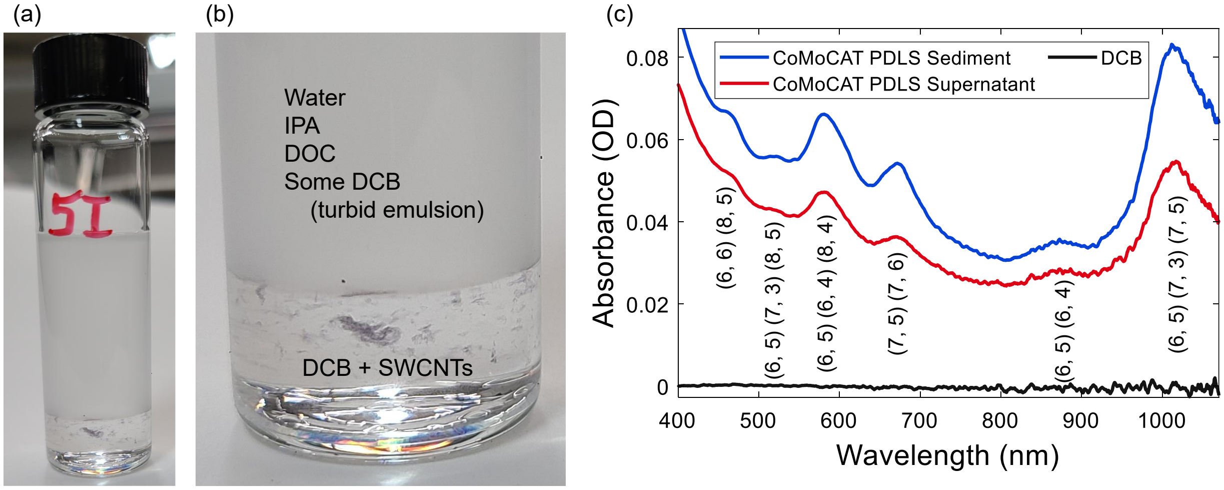

Figure S6: The solvent transfer process of CoMoCAT SWCNTs from aqueous surfactant suspension to DCB. (a) and (b) Image and close-up of the solvent transfer process 15 min after the vortexing step listed as step 7 in the solvent transfer procedure. A top turbid solution is visible in the upper phase and CNT flocculates are visible in the clear bottom phase of the solution. (c) Optical absorption spectra of the CoMoCAT SWCNTs after transfer to DCB. The presence of strong peaks is indicative of good individualization of the CNTs, even in a surfactant-free organic phase.

SWCNT Solvent Transfer

Single walled carbon nanotubes produced with the cobalt molybdenum catalyst (CoMoCAT) synthesis method[18] were first suspended in a solution of sodium deoxycholate (DOC). These CNTs were measured with analytical ultracentrifugation (AUC) and then transferred to DCB for measurement with the E-field-assisted dichroism-based length measurement technique. Dichlorobenzene was chosen as a solvent due to its low electrical conductivity and high voltage stability. To accomplish the solvent exchange, removal of the DOC surfactant shell was necessary, and was achieved in a manner similar to established methods,[19] an image of which is visible in Figs. S6(a) and S6(b). The transfer procedure was created specifically to minimize the DOC concentration in the final SWCNT suspension in DCB. To ensure good individualization of SWCNTs after the surfactant stripping and transfer to organic solvent, samples were examined after step 10 in a spectrometer, seen in Fig. S6(c). The presence of distinct peaks in the absorbance spectra are indicative of individualization.[20] Peaks from the absorbance spectrum are labeled with corresponding SWCNT chiralities.[14,20–27]

Solvent transfer procedure for CoMoCAT SWCNTs

1. Mix 10 µl SWCNT suspension in aqueous DOC with 5 ml deionized (DI) water.

2. Add 2 ml line 1 with 4 ml isopropanol (IPA) and 2 ml DCB. While the IPA strips the surfactant shell, the hydrophobic SWCNTs will naturally migrate to the DCB phase over the water-IPA phase.

3. Vortex 15 s to facilitate stripping of the surfactant shell and SWCNT migration.

4. Allow to rest for 24 h so that the phases become as distinct as possible.

5. Extract DCB phase and phase boundary including a small amount of aqueous phase to collect all CNTs at the interface.

6. Add 2 ml DI water and 4 ml isopropanol to further strip any associated surfactant and allow a phase for the disassociated surfactant to reside.

7. Vortex 15 s to facilitate removal of any additional surfactant.

8. Allow to rest for 24 h to allow for liquid phase separation to become distinct.

9. Extract DCB phase and phase boundary to ensure that any CNTs that have collected at the interface are acquired.

10. Place in vacuum chamber for 24 h to evaporate any residual water or IPA.

11. Dilute 200 µl sample in 6 ml DCB.

12. Bath sonicate 30 min.

13. Repeat steps 11 and 12 twice more to achieve a concentration appropriate for E-field-assisted length measurement.

References

(1) Lin, C.; Shan, J. W. Ensemble-averaged particle orientation and shear viscosity of single-wall-carbon-nanotube suspensions under shear and electric fields. *Physics of Fluids* **2010**, *22*, 022001.

(2) Brown, M. S.; Shan, J. W.; Lin, C.; Zimmermann, F. M. Electrical polarizability of carbon nanotubes in liquid suspension. *Applied Physics Letters* **2007**, *90*, 203108.

(3) Arenas-Guerrero, P.; Iglesias, G. R.; Delgado, Á. V.; Jiménez, M. L. Electric birefringence spectroscopy of montmorillonite particles. *Soft Matter* **2016**, *12*, 4923–4931.

(4) Arenas-Guerrero, P.; Delgado, Á. V.; Donovan, K. J.; Scott, K.; Bellini, T.; Mantegazza, F.; Jiménez, M. L. Determination of the size distribution of non-spherical nanoparticles by electric birefringence-based methods. *Scientific Reports* **2018**, *8*, 1–10.

(5) Jones, T. B. *Electromechanics of Particles*; Cambridge University Press: Cambridge, 1995.

(6) Landau, L. D.; Lifshitz, E. M. *Oxford University Press*; Pergammon Press: Oxford, 1960; Vol. 8.

(7) Islam, M. F.; Milkie, D. E.; Kane, C. L.; Yodh, A. G.; Kikkawa, J. M. Direct Measurement of the Polarized Optical Absorption Cross Section of Single-Wall Carbon Nanotubes. *Physical Review Letters* **2004**, *93*, 037404.

(8) Lefebvre, J.; Fraser, J. M.; Finnie, P.; Homma, Y. Photoluminescence from an individual single-walled carbon nanotube. *Physical Review B* **2004**, *69*, 1–5.

(9) Fagan, J. A.; Bajpai, V.; Bauer, B. J.; Hobbie, E. K. Anisotropic polarizability of isolated semiconducting single-wall carbon nanotubes in alternating electric fields. *Applied Physics Letters* **2007**, *91*.

(10) Bauer, B. J.; Fagan, J. A.; Hobbie, E. K.; Chun, J.; Bajpai, V. Chromatographic Fractionation of SWNT/DNA Dispersions with On-Line Multi-Angle Light Scattering. *The Journal of Physical Chemistry C* **2008**, *112*, 1842–1850.

(11) Liu, W.-W.; Chai, S.-P.; Mohamed, A. R.; Hashim, U. Synthesis and characterization of graphene and carbon nanotubes: A review on the past and recent developments. *Journal of Industrial and Engineering Chemistry* **2014**, *20*, 1171–1185.

(12) Lide, D. R. *CRC Handbook of Chemistry and Physics*, 84th ed.; CRC Press, 2003.

(13) Sanchez, S. R.; Bachilo, S. M.; Kadria-Vili, Y.; Lin, C. W.; Weisman, R. B. (n,m)-Specific Absorption Cross Sections of Single-Walled Carbon Nanotubes Measured by Variance Spectroscopy. *Nano Letters* **2016**, *16*, 6903–6909.

(14) Pfohl, M.; Tune, D. D.; Graf, A.; Zaumseil, J.; Krupke, R.; Flavel, B. S. Fitting Single-Walled Carbon Nanotube Optical Spectra. *ACS Omega* **2017**, *2*, 1163–1171.

(15) Meshot, E. R.; Park, S. J.; Buchsbaum, S. F.; Jue, M. L.; Kuykendall, T. R.; Schaible, E.; Bayu Aji, L. B.; Kucheyev, S. O.; Wu, K. J. J.; Fornasiero, F. High-yield growth kinetics and spatial mapping of single-walled carbon nanotube forests at wafer scale. *Carbon* **2020**, *159*, 236–246.

(16) Li, Y. et al. Autonomously Responsive Membranes for Chemical Warfare Protection. *Advanced Functional Materials* **2020**, *30*, 2000258.

(17) Park, S. J.; Moyer-Vanderburgh, K.; Buchsbaum, S. F.; Meshot, E. R.; Jue, M. L.; Wu, K. J.; Fornasiero, F. Synthesis of wafer-scale SWCNT forests with remarkably invariant structural properties in a bulk-diffusion-controlled kinetic regime. *Carbon* **2023**, *201*, 745–755.

(18) Resasco, D. E.; Alvarez, W. E.; Pompeo, F.; Balzano, L.; Herrera, J. E.; Kitiyanan, B.; Borgna, A. A scalable process for production of single-walled carbon nan-

otubes (SWNTs) by catalytic disproportionation of CO on a solid catalyst. *Journal of Nanoparticle Research* **2002**, *4*, 131–136.

(19) Cao, L.; Li, Y.; Liu, Y.; Zhao, J.; Nan, Z.; Xiao, W.; Qiu, S.; Kang, L.; Jin, H.; Li, Q. Iterative Strategy for Sorting Single-Chirality Single-Walled Carbon Nanotubes from Aqueous to Organic Systems. *ACS Nano* **2024**, *18*, 3783–3790.

(20) Jones, M.; Engtrakul, C.; Metzger, W. K.; Ellingson, R. J.; Nozik, A. J.; Heben, M. J.; Rumbles, G. Analysis of photoluminescence from solubilized single-walled carbon nanotubes. *Physical Review B - Condensed Matter and Materials Physics* **2005**, *71*, 1–9.

(21) Nair, N.; Usrey, M. L.; Kim, W. J.; Braatz, R. D.; Strano, M. S. Estimation of the (n,m) concentration distribution of single-walled carbon nanotubes from photoabsorption spectra. *Analytical Chemistry* **2006**, *78*, 7689–7696.

(22) Ghosh, S.; Bachilo, S. M.; Weisman, R. B. Advanced sorting of single-walled carbon nanotubes by nonlinear density-gradient ultracentrifugation. *Nature Nanotechnology* **2010**, *5*, 443–450.

(23) Ohmori, S.; Saito, T.; Tange, M.; Shukla, B.; Okazaki, T.; Yumura, M.; Iijima, S. Fundamental importance of background analysis in precise characterization of single-walled carbon nanotubes by optical absorption spectroscopy. *Journal of Physical Chemistry C* **2010**, *114*, 10077–10081.

(24) Streit, J. K.; Bachilo, S. M.; Ghosh, S.; Lin, C. W.; Weisman, R. B. Directly measured optical absorption cross sections for structure-selected single-walled carbon nanotubes. *Nano Letters* **2014**, *14*, 1530–1536.

(25) Ao, G.; Streit, J. K.; Fagan, J. A.; Zheng, M. Differentiating Left- and Right-Handed Carbon Nanotubes by DNA. *Journal of the American Chemical Society* **2016**, *138*, 16677–16685.

(26) Zheng, M. Sorting Carbon Nanotubes. *Topics in Current Chemistry* **2017**, *375*, 13.

(27) Yang, F.; Wang, M.; Zhang, D.; Yang, J.; Zheng, M.; Li, Y. Chirality Pure Carbon Nanotubes: Growth, Sorting, and Characterization. *Chemical Reviews* **2020**, *120*, 2693–2758.
